# The Impact of Prolonged Exposure Therapy on Posttraumatic Stress Disorder Symptom Clusters in Adults Maintained on Medications for Opioid Use Disorder

**DOI:** 10.1002/cpp.70199

**Published:** 2025-12-08

**Authors:** Jillian A. Giannini, Gary J. Badger, Rebecca Cole, Kelly R. Peck

**Affiliations:** ^1^ Department of Psychiatry University of Vermont Burlington Vermont USA; ^2^ Department of Psychology University of Vermont Burlington Vermont USA; ^3^ Department of Medical Biostatistics University of Vermont Burlington Vermont USA

**Keywords:** contingency management, opioid use disorder, posttraumatic stress disorder, prolonged exposure therapy

## Abstract

Although prolonged exposure (PE) therapy has shown promise for improving overall posttraumatic stress disorder (PTSD) severity in individuals receiving treatment for opioid use disorder (OUD), its effect on individual PTSD symptom clusters has not been examined in this population. Thus, we examined PTSD symptom clusters (intrusion, avoidance, negative alterations in cognitions and mood [NACM], arousal and reactivity) in 82 adults who participated in one of two randomized trials wherein participants with PTSD who were receiving medications for OUD (MOUD) were randomized to: MOUD treatment as usual (TAU; *n* = 27), prolonged exposure therapy (PE; *n* = 27) or PE plus financial incentives contingent upon session attendance (PE+; *n* = 28). PTSD symptoms were assessed using the Clinician Administered PTSD Scale for DSM‐5 at baseline and 4‐, 8‐ and 12‐week post‐randomization. We compared the effect of experimental condition on PTSD symptom clusters. PTSD symptom clusters improved significantly (*p*'s < 0.05) between baseline and week 12 (end of treatment) in all experimental conditions except for intrusion symptoms in TAU participants (*p* = 0.050). PE and PE+ participants reported greater improvements than TAU participants on intrusion symptoms (*p*'s < 0.05). PE+ participants also experienced greater improvements on avoidance symptoms than TAU participants (*p* < 0.001). PE and PE+ participants achieved comparable reductions on all symptom clusters. Furthermore, participants in all experimental conditions improved similarly on NACM and arousal and reactivity symptom clusters. These results support the putative mechanisms of PE and suggest that PE+ may be well suited for improving more trauma‐specific symptom clusters such as intrusion and avoidance in individuals receiving MOUD.

## Summary

- Both PE and PE+ were more effective than TAU in reducing intrusion symptoms.
- PE+ was more effective than TAU in reducing avoidance symptoms.
- PE+ may be a particularly useful treatment approach for patients at risk of poor attendance, including those who are highly avoidant or struggling with logistical barriers to PTSD treatment.

Posttraumatic stress disorder (PTSD) is particularly prevalent among those with opioid use disorder (OUD). Among those with OUD, the prevalence of current PTSD is approximately 18%, which is over four times higher than the general population (Santo et al. 2022). One explanation for the frequent co‐occurrence of PTSD and OUD is that some individuals may use opioids to cope with PTSD‐related physiological arousal or avoid trauma‐related stimuli, thereby reducing PTSD‐related distress (Smith et al. 2016). The short‐term relief opioids provide from these symptoms may negatively reinforce opioid use, increasing the likelihood of repeated use and eventually contributing to physical dependence, OUD and worsening mental health outcomes (Meshberg‐Cohen et al. 2021). Additionally, the neurobiological structures and pathways underlying PTSD and OUD (e.g., endogenous opioid system, hypothalamic–pituitary–adrenal axis) overlap substantially (Danovitch 2016; Shorter et al. 2015). When the two disorders co‐occur, this overlap may result in a mutual exacerbation and perpetuation of symptoms and require concurrent treatment of both PTSD and OUD symptoms.

Agonist medications for OUD (MOUD; e.g., buprenorphine and methadone) are efficacious for reducing illicit opioid use and other drug‐related risk behaviours (Mattick et al. 2014). However, untreated PTSD is associated with more severe mental health and psychosocial problems among individuals receiving MOUD treatment (Meshberg‐Cohen et al. 2021). This more severe clinical profile may undermine MOUD treatment efficacy and retention (Hien et al. 2009; Peirce et al. 2016; Schiff et al. 2010). The concurrent delivery of evidence‐based PTSD treatment may be beneficial for individuals with PTSD who are receiving MOUD as these interventions are associated with improvements in PTSD‐related mental health symptoms and increased MOUD treatment engagement (Meshberg‐Cohen et al. 2019; Schacht et al. 2017).

Prolonged exposure therapy (PE) is an evidence‐based PTSD treatment that has shown promise for improving PTSD symptoms among those receiving treatment for OUD (Peck et al. 2018; Schacht et al. 2017). However, poor treatment attendance may limit the efficacy of PE. In the general population, one in three participants do not complete PE (Imel et al. 2013; Varker et al. 2021). People with co‐occurring PTSD and substance use disorders (SUDs) may be even more likely to discontinue PE prematurely, with some studies reporting dropout rates as high as 62% (Belleau et al. 2017). Indeed, approximately 25% of participants with concurrent PTSD and SUD discontinue PE before attending a single session (Coffey et al. 2006; Foa et al. 2013; Mills et al. 2012). As a result, many patients discontinue treatment before beginning exposure, the active component of treatment (Brady et al. 2001; Mills et al. 2012; Sannibale et al. 2013).

Behavioural economic approaches may be well suited for improving PE attendance. In two recent randomized trials, adults with a current diagnosis of PTSD who were receiving buprenorphine or methadone treatment for OUD were randomized to receive: (a) continued MOUD treatment as usual (TAU), (b) PE or (c) PE with financial incentives delivered contingent upon PE session attendance (PE+; Peck et al. 2023; Peck et al. 2025). The aim of these studies was to evaluate whether PE+ participants would attend more therapy sessions than PE participants and, as a result, achieve greater improvements in PTSD symptoms compared to PE or TAU participants. In both trials, participants who received PE+ attended significantly more therapy sessions than participants who received PE. Although the two PE groups did not differ in either study on PTSD symptom severity as measured by the Clinician Administered PTSD Scale for DSM‐5 (CAPS‐5; Weathers et al. 2013a), there was promising support for the efficacy of PE+ compared to TAU. In the initial pilot trial, PE+ participants achieved significantly greater reductions in PTSD symptoms than TAU participants (mean difference = 10.9, *d* = 1.34, *p* = 0.046; Peck et al. 2023). A similar pattern was observed in the subsequent larger randomized trial. However, the difference in reductions in PTSD symptoms between PE+ and TAU conditions did not reach statistical significance (mean difference = 7.0, *d* = 0.84, *p* = 0.06; Peck et al. 2025). Although PE was efficacious for reducing total PTSD severity in these trials, examination of individual PTSD symptom clusters may provide important theoretically and clinically relevant information related to mechanisms of change.

The active components of PE are imaginal and in vivo (real life) exposure exercises that allow patients to confront trauma‐related memories and real‐life reminders that have been previously avoided but are not inherently harmful (Foa et al. 2019). Accordingly, PE may be particularly efficacious for reducing avoidance symptoms. PE may also reduce intrusion, arousal and reactivity symptoms through fear reduction that is expected to occur with emotional processing. In the general population, these expectations have been partially supported. PE has outperformed eye‐movement desensitization and reprocessing and relaxation training for improving avoidance and intrusion symptoms (Taylor et al. 2003). Furthermore, PE had a unique effect of reducing clinician‐rated avoidance and numbing symptoms (now called negative alterations in cognitions and mood [NACM] in DSM‐5) above and beyond present‐centred therapy (Schnurr and Lunney 2015). In these studies, PE did not have a unique effect on arousal and reactivity symptoms, which may be particularly elevated among those with OUD (Tull et al. 2010).

There is promising support for the efficacy of PE for improving overall PTSD severity among individuals receiving MOUD treatment. However, the effect of PE on individual PTSD symptom clusters in this vulnerable population has yet to be investigated. This question is particularly important given that opioid agonist medications themselves may be associated with significant improvements in PTSD symptoms, with some studies showing improvements in physiological arousal and mood symptoms that are not directly targeted by PE (Dean et al. 2004; Lake et al. 2019; Shorter et al. 2015). Determining whether PE outperforms MOUD alone for improving specific PTSD symptom clusters will yield valuable information related to mechanisms of change as well as specific PE‐related benefits for this population. Accordingly, this secondary analysis was conducted to examine the effects of PE on individual PTSD symptom clusters in individuals receiving MOUD treatment. Based on the findings of prior studies (Taylor et al. 2003; Schnurr and Lunney 2015), we hypothesized that participants in the PE and PE+ conditions would report greater improvements in avoidance and intrusion symptoms than those who receive TAU.

## 1 Method

### 1.1 Participants and Procedure

Participants were 82 adults (≥ 18 years old) who participated in one of two recent 12‐week randomized trials (Peck et al. 2023; Peck et al. 2025). All participants met DSM‐5 criteria for PTSD based on the CAPS‐5 (Weathers et al. 2013a) and were maintained on a stable methadone or buprenorphine dose for ≥ 1 month prior to enrolment. Exclusion criteria were active delusions, hallucinations, mania or imminent risk of suicide as assessed by the Mini International Neuropsychiatric Interview (MINI; Sheehan et al. 1997) or medical conditions that could interfere with consent or study participation. Those currently enrolled in evidence‐based PTSD treatment were also excluded.

Participants were recruited using advertisements distributed throughout the community, to local MOUD treatment providers and online. Participants provided written informed consent, signed a release of information permitting study staff to confirm MOUD treatment enrolment, dose and time on that dose and completed an initial assessment to determine study eligibility. Eligible participants were randomly assigned to either: (a) TAU (*n* = 27), (b) PE (*n* = 27) or (c) PE+ (*n* = 28). Following intake, assessment visits were conducted at 4–8 and 12 weeks after randomization. Staff who conducted follow‐up assessments were not blinded to experimental condition. All study procedures were approved by the university's institutional review board.

### 1.2 Treatment Conditions

#### 1.2.1 TAU

Participants assigned to the TAU condition continued to receive MOUD treatment from their current provider and completed monthly study assessments but did not receive PTSD treatment as part of study participation.

#### 1.2.2 PE

In addition to receiving continued MOUD treatment and completing monthly assessments, PE participants received up to 12 60‐min sessions of PE. PE included psychoeducation about PTSD, breathing retraining and repeated imaginal and in vivo (real life) exposure exercises to reduce PTSD symptoms. Although this protocol was largely based on conventional PE (Foa et al. 2019), several adaptations were made. In addition to limiting the duration of sessions to 60 min, participants received psychoeducation regarding the relationship between PTSD and OUD and weekly check‐ins about substance use and were permitted to complete therapy sessions in person or via a telehealth platform.

#### 1.2.3 PE+

Participants assigned to the PE+ condition received the procedures described for the PE condition plus financial incentives delivered contingent upon completion of PE sessions. Participants earned gift cards for attending scheduled PE appointments. Gift cards were delivered immediately following completion of each session. The initial session was worth $20, and each consecutive attended session increased the incentive amount by $5. In addition, participants received a $50 bonus for every two consecutive sessions attended and a $100 bonus if they completed all 12 sessions. Missed sessions earned no incentives and reset the incentive value for the next attended session back to the initial $20 value. However, two consecutive attended PE sessions following a reset returned the incentive value back to the value immediately prior to the missed appointment. Overall, participants who attended every session could earn a maximum of $920.

### 1.3 Measures

#### 1.3.1 Demographic Information and Drug Use History

A staff‐administered questionnaire was administered at intake that assessed basic demographic information and drug use and treatment history, including participant age, sex, ethnicity, MOUD medication and duration of MOUD treatment.

#### 1.3.2 Co‐Occurring Mental Health Disorders

The MINI is a brief structured diagnostic interview that was administered at intake to assess for major depressive disorder, suicidality, bipolar disorders and related disorders, panic disorder, agoraphobia, social anxiety disorder, obsessive‐compulsive disorder, alcohol use disorder, substance use disorder, psychotic disorders, anorexia nervosa, bulimia nervosa and generalized anxiety disorder. The MINI is a valid and reliable measure of these disorders (Sheehan et al. 1997).

#### 1.3.3 Lifetime Trauma History

Trauma history was assessed at intake using the Life Events Checklist for DSM‐5 (LEC‐5; Weathers et al. 2013b). The LEC‐5 is a 17‐item self‐report measure that assesses exposure to 16 different types of potentially traumatic events (e.g., natural disaster, fire or explosion, physical assault), plus an additional open category for any other very stressful event not captured in other items. Participants were asked to indicate whether they had experienced, witnessed, learned about, or were never exposed to each type of traumatic event.

#### 1.3.4 PTSD Symptoms

PTSD symptoms were assessed using the CAPS‐5 (Weathers et al. 2013a) at intake and 4‐, 8‐ and 12‐week postrandomization. The CAPS‐5 is a well‐established and widely used 30‐item structured diagnostic clinical interview designed to assess DSM‐defined PTSD symptoms. In addition to establishing a Criterion A index trauma, the CAPS‐5 assessed the intensity and frequency of each of the 20 DSM‐5 PTSD symptoms (Weathers et al. 2018). On the CAPS‐5, interviewers provided a single symptom severity score for each individual item ranging from 0 to 4 (0 = *Absent* to 4 = *Extreme/Incapacitating*) with total PTSD symptom severity scores ranging from 0 to 80. In addition to the total severity score, symptom clusters were measured independently within sections corresponding to Criterion B (intrusion cluster consisting of five items and resulting in an intrusion severity score ranging from 0 to 20), Criterion C (avoidance cluster consisting of two symptoms and resulting in an avoidance severity score ranging from 0 to 8), Criterion D (NACM cluster consisting of seven symptoms and resulting in a NACM severity score ranging from 0 to 28) and Criterion E (arousal and reactivity cluster consisting of six symptoms and resulting in an arousal and reactivity severity score ranging from 0 to 24).

### 1.4 Data Analysis

Participants in TAU, PE and PE+ were compared on baseline demographic and clinical characteristics using *t* tests for continuous measures and chi‐square or Fisher's exact tests for categorical measures. Outcome measures consisted of the four CAPS‐5 cluster scores (avoidance, intrusion, NACM and arousal and reactivity). Mixed model repeated measures analyses were used to compare temporal changes between TAU, PE and PE+ groups on each of the cluster severity scores assessed at intake and 4‐, 8‐ and 12‐week post‐randomization. The model included one across‐subject fixed factor, experimental condition and one within‐subject repeated fixed factor, time. Subject effects represented a random factor nested within condition. All means presented represent least square means derived from mixed model repeated measures analyses, which account for missing data due to incomplete follow‐up. Linear contrasts were used to compare changes from intake to the end of treatment (week 12) between and within TAU, PE and PE+ groups. Consistent with an intention‐to‐treat approach, repeated measures analyses included all randomized participants independent of early dropout. Analyses were conducted using SAS Statistical Software, V9.4 (SAS Institute, Cary, NC, USA), with statistical significance based on *p* < 0.05.

## 2 Results

### 2.1 Baseline Demographic and Clinical Characteristics

Table 1 presents the demographic and clinical characteristics of the study sample. Participants were 39 years old on average and most (67.9%) were female. Most participants were receiving buprenorphine (63.4%) and had been receiving MOUD treatment for an average of 4.5 years. With respect to the total number of PE sessions attended, PE+ participants attended significantly more (10.5; SD = 2.0) therapy sessions than PE participants (4.0; SD = 3.3; *p* < 0.001). The three groups were similar across most other demographic and clinical characteristics.

**TABLE 1:** Demographic and Clinical Characteristics.

	Total (*N* = 82)	TAU (*n* = 27)	PE (*n* = 27)	PE+ (*n* = 28)
Age	39.0 (9.7)	42.9 (10.1)	37.6 (9.0)	36.7 (9.1)
Female, *n* (%)	55 (67.9)	19 (70.4)	17 (63.0)	19 (70.4)
White, *n* (%)	74 (92.5)	25 (92.6)	22 (88.0)	27 (96.4)
Time on MOUD^a^	3.6 (0.9, 6.0)	3.2 (0.7, 7.0)	3.7 (1.0, 7.0)	4.0 (0.9, 5.0)
Prescribed buprenorphine, *n* (%)	52 (63.4)	18 (66.7)	18 (66.7)	16 (57.1)
CAPS‐5 PTSD severity	39.5 (8.2)	37.4 (8.9)	40.2 (8.9)	40.8 (6.7)
Index trauma, *n* (%)				
Sexual assault	33 (40.2)	10 (37.0)	11 (40.7)	12 (42.9)
Witnessed injury or death	17 (20.7)	6 (22.2)	6 (22.2)	5 (17.9)
Physical assault	13 (15.9)	7 (25.9)	3 (11.1)	3 (10.7)
Accident	9 (11.0)	2 (7.4)	4 (14.8)	3 (10.7)
Learned about death or injury	5 (6.1)	2 (7.4)	0 (0)	3 (10.7)
Other	3 (3.7)	0 (0)	2 (7.4)	1 (3.6)
Disaster	1 (1.2)	0 (0)	0 (0)	1 (3.6)
Combat	1 (1.2)	0 (0)	1 (3.7)	0 (0)
Current anxiety or depressive disorder^b^, *n* (%)	68 (82.9)	20 (74.1)	23 (85.2)	25 (89.3)
PE sessions attended	7.3 (4.2)	—	4.0 (3.3)	10.5 (2.0)

*Note:* Values represent mean (SD), unless otherwise indicated.

^a^ Data represents median (interquartile range).

^b^ Current anxiety and depressive disorders assessed via the Mini‐International Neuropsychiatric Interview.

Abbreviations: CAPS‐5, Clinician Administered PTSD scale for DSM‐5; MOUD, medications for opioid use disorder; PE, prolonged exposure therapy; PE+, PE with financial incentives delivered contingent upon PE session attendance; PTSD, posttraumatic stress disorder; TAU, continued MOUD treatment as usual.

### 2.2 Intrusion Cluster Severity

**FIGURE 1:**
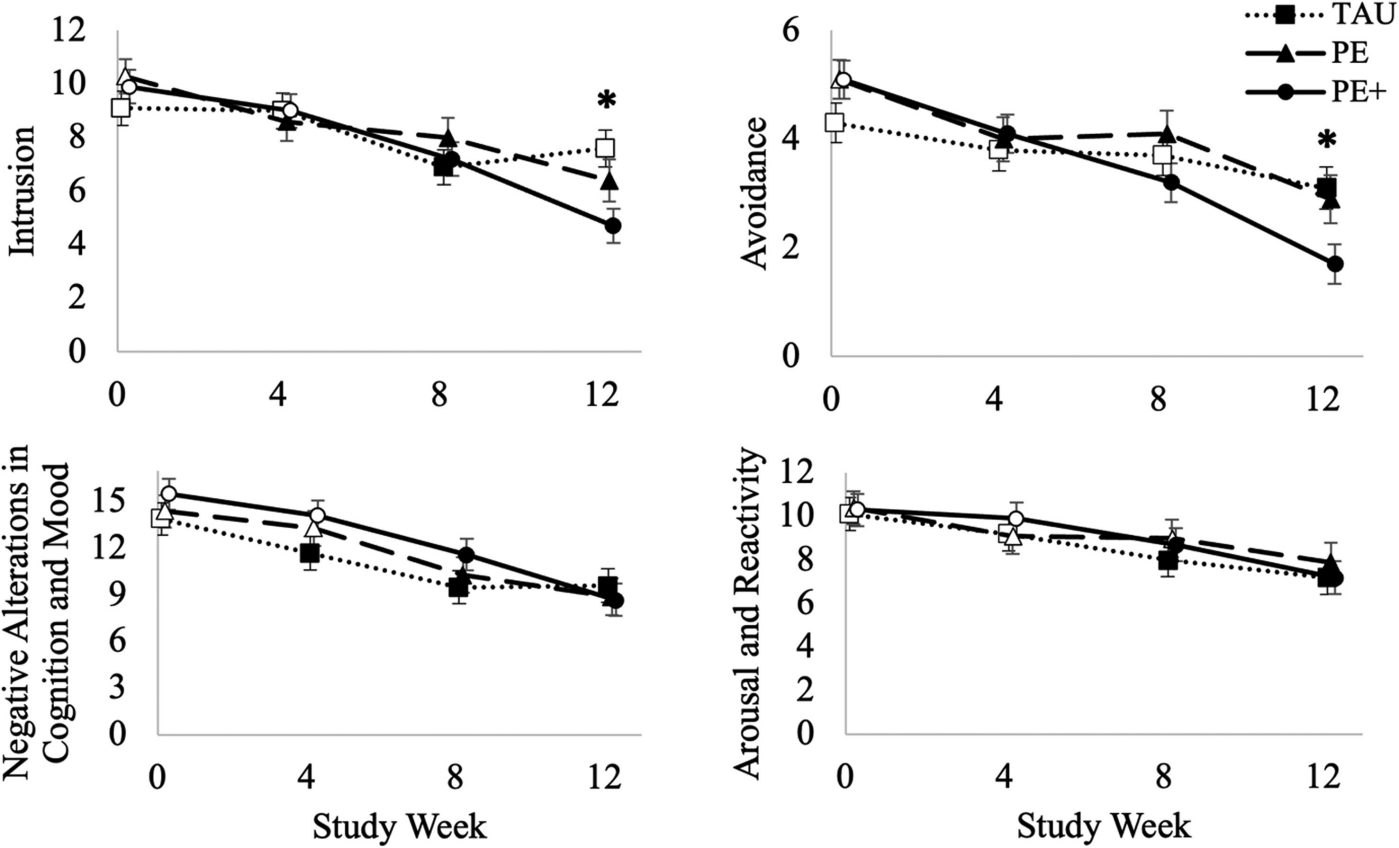
Changes over time in CAPS‐5 intrusion, avoidance, negative alterations in cognitions and mood (NACM), and arousal and reactivity symptom severity for participants randomized to MOUD treatment as usual (TAU), prolonged exposure therapy (PE) or PE with financial incentives contingent upon PE session attendance (PE+). Error bars represent standard errors associated with least square means. Solid symbols indicate a significant within‐group change from intake; asterisks indicate that the change from intake to assessment timepoint significantly differed between groups (*p* < 0.05).

With respect to changes in intrusion symptoms, temporal changes were significantly different across groups, *F*(6, 209) = 2.77, *p* = 0.013 (Figure 1, top left panel). Specifically, PE+ participants, *F*(1, 206) = 11.98, *p* < 0.001, and PE participants, *F*(1, 215) = 4.64, *p* = 0.032, demonstrated greater improvements from baseline to the end of treatment (week 12) in intrusion symptoms than TAU participants. PE, *t*(221) = 4.66, *p* < 0.001, and PE+, *t*(204) = 7.08, *p* < 0.001, participants had a significant decrease in intrusion severity between intake and week 12. Meanwhile, reductions in TAU participants' intrusion symptoms between intake and week 12 fell just short of the threshold for statistical significance, *t*(208) = 1.97, *p* = 0.050. Those in PE and PE+ experienced similar reductions in intrusion symptoms, *F*(1, 214) = 1.13, *p* = 0.290.

### 2.3 Avoidance Cluster Severity

For avoidance symptoms, changes over time were significantly different across groups, *F*(6, 211) = 2.56, *p* = 0.020 (Figure 1, top right panel). PE+ participants experienced greater improvements in their avoidance symptoms than TAU participants, *F*(1, 208) = 12.46, *p* < 0.001. However, participants in the TAU, *t*(210) = 2.82, *p* = 0.005, PE, *t*(224) = 4.63, *p* < 0.001, and PE+, *t*(206) = 8.07, *p* < 0.001, groups all reported significant improvements in their avoidance symptoms between intake and week 12. Although participants in the PE+ group reported numerically greater reductions in avoidance symptoms compared to PE participants, differences between these two experimental conditions fell just short of the threshold for statistical significance, *F*(1, 216) = 3.04, *p* = 0.083.

### 2.4 Negative Alterations in Cognitions and Mood Cluster Severity

With respect to changes in NACM symptoms, a significant main effect of time was observed, *F*(3, 206) = 32.45, *p* < 0.001, with all three groups demonstrating significant improvements in NACM symptoms over the 12‐week intervention period. There were no differences in these changes between groups (group × time interaction: *F*(6, 206) = 1.08, *p* = 0.375; Figure 1, bottom left panel).

### 2.5 Arousal and Reactivity Cluster Severity

A significant main effect of time was observed for arousal and reactivity symptoms, *F*(3, 206) = 13.72, *p* < 0.001, with all three experimental groups experiencing significant improvements in their arousal and reactivity symptoms with no differences between groups (group × time interaction: *F*(6, 206) = 0.37, *p* = 0.899; Figure 1, bottom right panel).

## 3 Discussion

The current study examined the effect of PE on PTSD symptom clusters among individuals receiving MOUD treatment. Although prior studies have examined PE's effect on PTSD symptom clusters in those without substance use disorders, the present study is the first to our knowledge to extend that work to individuals receiving treatment for OUD. Our findings indicate that PE and PE+ participants achieved greater improvements in the severity of intrusion symptoms compared to TAU participants. These findings are consistent with prior studies, which found unique effects of PE for improving intrusion symptoms (Taylor et al. 2003). Furthermore, the effect of PE on improvements in intrusion symptoms provides additional support for the theoretical foundations of PE. Specifically, imaginal exposure may allow patients enrolled in MOUD treatment to confront trauma‐related thoughts and feelings that were previously avoided, resulting in gradual reductions in fear associated with the traumatic memory and other trauma cues.

As hypothesized, PE+ participants reported significantly greater reductions in avoidance symptoms than TAU participants. This finding was expected given PE's emphasis on confronting safe but previously avoided trauma reminders via imaginal and in vivo exposure. Although these findings align with the results of prior studies indicating PE's unique effects for improving avoidance symptoms compared to other therapies (Taylor et al. 2003; Schnurr and Lunney 2015), it is noteworthy that the PE and TAU conditions did not differ significantly in terms of avoidance symptom improvement. One possible explanation for this finding is that PE participants attended far fewer PE sessions on average compared to PE+ participants (4.0 vs. 10.5) and did not confront trauma‐related thoughts, feelings and situations with sufficient frequency to achieve significant reductions in avoidance symptoms compared to TAU participants.

There were no significant group differences in NACM or arousal and reactivity symptom clusters. Although exposure therapy may address these symptoms by introducing new information that may disconfirm unhelpful, negative cognitions and promoting behavioural activation, it is possible that other treatments that specifically target negative cognitions and mood (e.g., cognitive behavioural therapy for depression) may be beneficial for some patients even after a successful course of PE. Furthermore, the findings of this study are supported by prior work, which has shown that arousal and reactivity symptoms are less responsive to PE (Taylor et al. 2003; Horesh et al. 2017; Schnurr and Lunney 2015). Thus, these symptoms may be difficult to change without interventions that specifically target these symptoms (e.g., relaxation training; Taylor et al. 2003).

The similarities in efficacy between PE and PE+ also warrant mention. Although PE+ participants attended significantly more sessions than PE participants, the two PE groups did not differ in improvements on any symptom clusters. Despite these similarities, it is notable that while both PE groups achieved significant improvements in intrusion symptoms compared to TAU, only PE+ participants achieved improvements in avoidance symptoms above and beyond TAU. Although the general absence of differences between the PE and PE+ groups may be attributable to limited statistical power, these results may also suggest that intrusion symptoms respond earlier in treatment while PE participants were still attending therapy sessions. In contrast, although PE effectively targets avoidance symptoms, these effects may not be realized until later in treatment when in vivo and imaginal exposure exercises are underway, and many PE participants had discontinued treatment. This conclusion is supported by prior work showing changes in intrusion symptoms early in exposure treatment (Maples‐Keller et al. 2017) and stands in contrast to prior evidence suggesting that PE tends to improve avoidance symptoms more quickly than intrusion symptoms (Taylor et al. 2003; Moshier et al. 2024). Therefore, additional research is needed with larger samples to clarify the temporal order of PE‐related changes in PTSD symptom clusters among individuals receiving MOUD treatment and to determine whether the clinical benefits of PE+ above and beyond PE justify the substantial financial costs.

It is also notable that TAU participants demonstrated significant improvements across PTSD symptom clusters except for intrusion symptoms. These findings are consistent with previous studies reporting symptom reductions in TAU control groups (Torchalla et al. 2012; van Dam et al. 2012). These reductions may be attributable to assessment reactivity or other unmeasured factors. However, there is some evidence that MOUD treatment may be associated with improvements in PTSD symptoms (Dean et al. 2004; Lake et al. 2019). These MOUD‐related improvements in PTSD symptoms may at least partially account for the absence of between‐group differences on the NACM and arousal and reactivity symptom clusters. Future studies may benefit from comparing the effects of PE on PTSD symptom clusters in a larger sample of individuals receiving MOUD treatment.

In terms of clinical implications, given that both PE groups reported significant reductions in intrusion symptoms, PE may be a particularly helpful treatment approach for individuals receiving MOUD treatment who are experiencing pronounced intrusion symptoms. Although it may be cost‐prohibitive to implement PE+ with all patients with PTSD who are receiving MOUD, there are clear clinical applications for this novel treatment approach. Given that this study demonstrated that participants who received PE+ were more likely to attend therapy sessions compared to PE participants, PE+ may be particularly useful for those impacted by common barriers to treatment access (e.g., limited financial resources, unreliable transportation) and at risk of poor engagement or dropout. Moreover, because PE+ participants experienced greater improvements in avoidance symptoms compared to TAU participants, PE+ may also be a helpful treatment approach for patients who are highly avoidant and more likely to discontinue treatment once exposure exercises begin. Alternatively, treatment providers may consider a stepped care approach in which attendance‐contingent financial incentives could be incorporated to improve therapy attendance for patients who struggle to consistently attend PE sessions. Research examining baseline predictors of response to PE versus PE+ could further inform personalized treatment and stepped care approaches.

This study has several strengths and extends the findings of prior studies in several ways. First, the present study used the CAPS‐5, which is a well‐established and widely used measure of PTSD, increasing confidence in the reported findings. Second, unlike many of the previous studies that assessed the effect of PE on DSM‐IV‐defined PTSD symptoms (Taylor et al. 2003; Schnurr and Lunney 2015), we assessed DSM‐5‐defined PTSD symptoms. Finally, to our knowledge, this is the first study to evaluate the effect of PE on PTSD symptom clusters in individuals receiving treatment for OUD. Despite these strengths, several limitations should be noted. First, our sample was largely non‐Hispanic White. Therefore, our findings may not generalize to other populations. Second, our sample had been receiving their MOUD for more than 4 years on average. Accordingly, changes in PTSD symptoms immediately following the initiation of MOUD were not captured. Future studies could evaluate the efficacy of PE for reducing PTSD symptoms in individuals initiating MOUD treatment to better disentangle the specific effects of PE from the improvements in mental health and psychosocial functioning that are often seen with MOUD treatment. Third, although these findings are consistent with the interpretation that PE+ participants achieved greater improvements in intrusion and avoidance symptoms than TAU participants because they attended more PE sessions and received a sufficient ‘dose’ of treatment, additional research is needed to better understand the mechanisms by which PE+ improves specific PTSD symptom clusters and the complex relationship between PE attendance and PTSD outcomes. Specifically, this research should examine PE attendance collapsed across experimental conditions to determine whether increased PE session attendance leads to improved symptom reduction.

In summary, to our knowledge, this was the first study to investigate the impact of PE on PTSD symptom clusters among individuals receiving MOUD. Although those with concurrent PTSD and OUD may present with exacerbated PTSD symptom profiles (Tull et al. 2010), these results demonstrate that PE+ participants, who received financial incentives for attending PE sessions and attended more therapy sessions than PE participants, demonstrated greater improvements in avoidance and intrusion symptom clusters compared to TAU participants. In addition, results show that participants who attend even a few sessions of PE may experience significantly greater improvements in intrusion symptoms than those who receive MOUD alone. However, all three groups achieved comparable improvements in NACM and arousal and reactivity clusters. Consistent with the findings of studies conducted among individuals without substance use disorders (Taylor et al. 2003; Schnurr and Lunney 2015), our results support the putative mechanisms of PE and suggest that PE+ may be particularly well suited for improving more trauma‐specific symptom clusters such as intrusion and avoidance.

## Funding

This work was supported by the National Institute on Drug Abuse (5T32DA007242 & R01DA057308) and the National Institute of General Medical Sciences (P20GM103644). The clinical trial number associated with data used in this analysis is NCT04104022.

## Data Availability

The data that support the findings of this study are available from the corresponding author upon reasonable request.
